# The Beat

**DOI:** 10.1289/ehp.121-a47

**Published:** 2013-02-01

**Authors:** Erin E. Dooley

**Affiliations:** Erin E. Dooley, MA, is a staff writer for *EHP*.

## A New Lighting Alternative?

Wake Forest University engineers are using multiwalled carbon nanotubes to enhance the brightness of field-induced polymer electroluminescent technology, potentially offering a step forward in the search for safe, pleasing, high-efficiency lighting.^1^ In this technology, moldable polymer matrix emits light when exposed to an electrical current. It could eventually yield high-efficiency lights without the mercury vapor of compact fluorescent lamps or the bluish tint of some fluorescents and LEDs, which has been linked with circadian rhythm disruption.^2^

**Figure:**
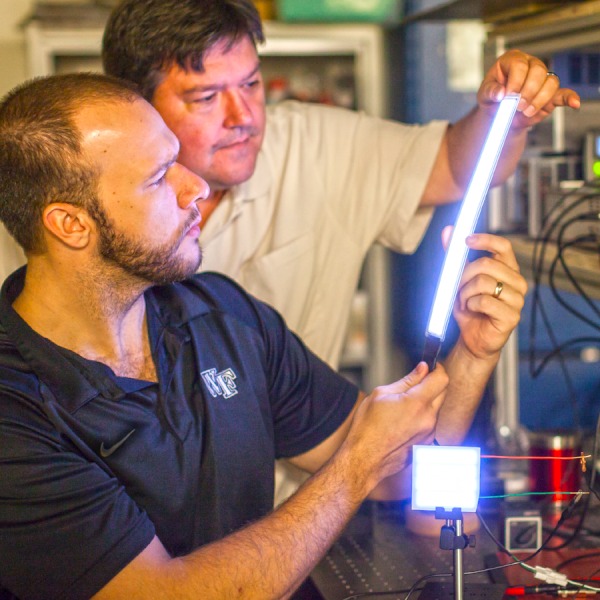
Wake Forest University engineers with the experimental light. Ken Bennett, Wake Forest University photographer

## Ceramic Cookstoves May Not Improve Child Pneumonia Rates

Pneumonia is the leading cause of death among children under age 5 in developing countries. Exposure to smoke from fires for cooking and heating is a major risk factor for pneumonia, and cleaner-burning cookstoves are being explored as an intervention. But a new study of locally produced ceramic cookstoves (*upesi jikos*) in Kenya suggests that “cleaner” isn’t always clean enough when it comes to cookstoves.^3^ Although *upesi jikos* were linked with fewer respiratory symptoms, there was no appreciable reduction in pneumonia among babies in households using the stoves compared with babies from households that cooked with traditional three-stone firepits. The authors conclude there is an urgent need for “further research into cookstove design, improved methodology for measuring PM_2.5_ exposures, and adequately-powered health impact trials.”

## EPA Revises Total Coliform Rule

Coliform bacteria in water are a marker for fecal contamination, which can cause gastroenteritis. This unpleasant but generally nonserious illness can lead to further health problems in vulnerable populations. But not all coliforms are fecal coliforms, so in December 2012 the U.S. EPA revised its Total Coliform Rule for drinking water to establish a maximum contaminant level specifically for *Escherichia coli*.^4^ With this more targeted test for fecal contamination, the public must be notified anytime drinking water is found to exceed this level. Noncompliant water systems also must conduct an inspection to locate and correct sources of fecal contamination.

**Figure:**
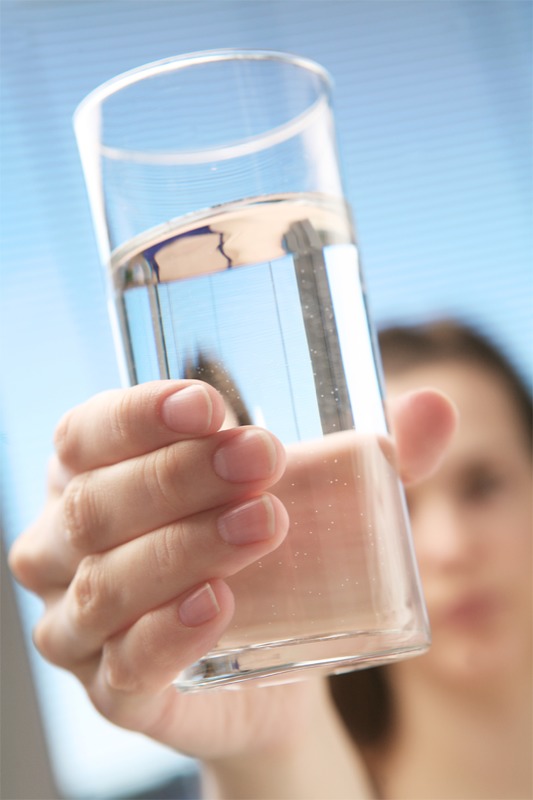
The EPA has mandated more targeted testing for fecal contamination of drinking water sources. Bomers et al

## Sound Alerts for Quiet Vehicles

The National Highway Traffic Safety Administration has proposed new rules for “alert sounds” that, when added to quiet electric and hybrid vehicles, will warn pedestrians and cyclists of their presence.^5^ The proposed rules address concerns that such alerts would increase community noise pollution^6^ with specifications designed to minimize noise impact while providing ample warning. Under the proposed rules, vehicles must be audible under a wide range of usual street and urban noises when the vehicle is moving slower than 18 mph. The agency estimates that adding alerts will prevent 2,800 pedestrian and cyclist injuries over the life of the 2016 model year fleet.^5^

## NYC Fish Is Often Mislabeled

In its latest study on the occurrence of mislabeled fish in stores and restaurants, the nonprofit Oceana found that more than a third of the fish sampled in New York City had been mislabeled.^7^ Tilefish, which is not recommended for small children or pregnant or nursing women because of its high mercury content, was sold as both halibut and red snapper at one grocery store, and 94% of samples of “white tuna” from New York sushi restaurants was actually escolar, which can cause explosive diarrhea due to its high content of wax esters. In all, 58% of the restaurants and stores sampled sold mislabeled fish.

**Figure:**
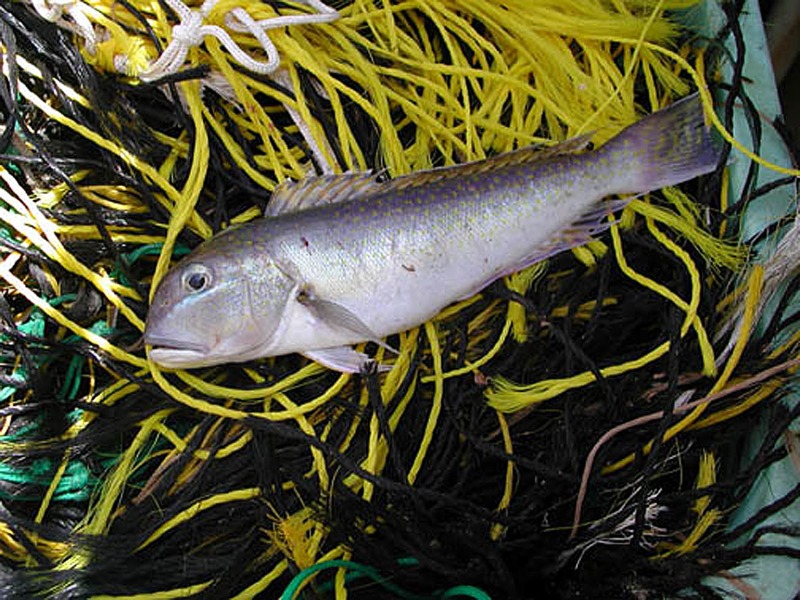
Some New York shoppers were sold tilefish, which can be high in mercury, instead of the safer species they thought they were buying. Bomers et al
